# Exploration of Wild Edible Plants Used as Food by Gaddis-A Tribal Community of the Western Himalaya

**DOI:** 10.1155/2020/6280153

**Published:** 2020-02-21

**Authors:** Arti Thakur, Somvir Singh, Sunil Puri

**Affiliations:** School of Biological and Environmental Sciences, Faculty of Basic Sciences, Shoolini University of Biotechnology and Management Sciences, Solan 173229, Himachal Pradesh, India

## Abstract

A survey of wild edible plants of Gaddi tribes of Himachal Pradesh was carried out in Chamba and Kangra districts of Himachal Pradesh located in Western Himalayas. The inhabitants subsisted primarily on pastoralism and agriculture and have traditional knowledge on wild edible plants. A total of 49 edible plants belonging to 24 families were recorded in the study area. These were commonly used as vegetables, fruits, spices, and chutney. Nearly half of the species belong to Polygonaceae and Rosaceae families. Herbs, shrubs, climbers, and trees form the habit of these plants. The highest proportion of edible species were herbs (29) followed by trees (10), shrubs (8), climber (1), and *Morchella esculenta* (fungi) (1).

## 1. Introduction

India being developing country and land of villages, elevated rates of poverty persist among rural communities, inspite of green revolution. The high economic growth has failed to improve food security in the mountainous regions of the Himalayas [1]. Fortunately, the Himalayas are known for rich biodiversity, especially for wild edible plants, which play an important role in meeting food demands. The rural inhabitants who mainly comprise of herders, shepherds, or other marginalized population use wild plants frequently for their livelihood [2]. The need is to understand the biodiversity for resource management of the Himalaya. This requires documentation of resources through ethnobotanical studies and for the conservation and utilization of resources. Moreover, the transmission of traditional knowledge from older to younger generations no longer exists; [3] thus, it is important to document the resources, especially of wild edible plants.

The Gaddis, the nomadic sheep and goat herders, are one of the most important migratory tribes of the Himalaya [4]. In Himachal Pradesh, Gaddis belongs to the Kangra and Chamba districts who move their livestock from one grazing ground to another in a seasonal cycle, to plains in winter and hilltops in summer. The livelihood of Gaddis depends on animal products and natural resources, such as plants and forests [3, 5]. Traditional knowledge of plants and their properties has always been transmitted from generation to generation through the natural course of everyday life. Transmission of traditional knowledge between the older and younger generation is no longer exists that's why the continuation of traditional knowledge is endangered [6]. Wild plants are richer in minerals compared to cultivated ones, and these plants may satisfy the daily human need for elementary nutrition sources, particularly those of Vitamin C and A, and for some minerals according to WHO regulation [7]. Wild edible plants provide vegetables, fruits, staple food, and spices for indigenous people and are the main source of food. These plants play an important role in the development of new crops through domestication, giving rise to cultivated food plants and strengthening local food security [8–11]. Consumption of wild plants has been a way of life for many rural populations throughout the world. Use of wild edible plants is an ancient tradition that has been increasingly neglected [12]. Due to socioeconomic changes, indigenous knowledge has been gradually destroyed by globalization and modern lifestyles [13, 14]. At the same time, the loss of indigenous knowledge has been discovered to be one of the major threats to the sustainability of biological diversity [15]. The aim of present study is to document wild edible plants used by Gaddis of Chamba and Kangra regions located in Himachal Pradesh, a mountainous state in the Himalaya. In addition, the present study is initiated from the remote area, with an aim to document the knowledge on the utilization of wild edible plants.

## 2. Materials and Methods

**Figure 1:**
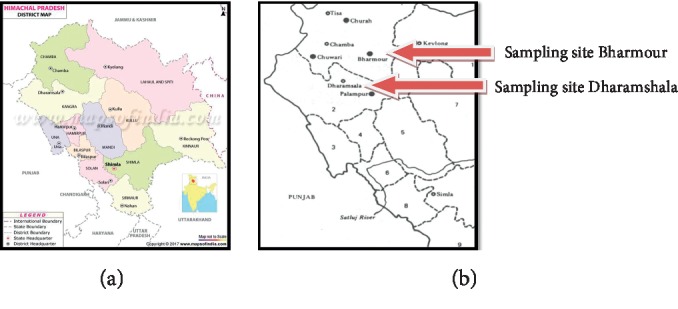
Map of Himachal Pradesh showing study sites.

The study area comprises of Chamba and Kangra districts located in the state of Himachal Pradesh. Extensive field surveys were made during 2017 and 2018 in Bharmour of District Chamba and Dharamshala (Khanyara) of district Kangra (Figure 1). Bharmour region lies between 1500 m to 3700 m amsl and extends 32°11′35″ to 32°41′54″N latitude 76°31′35″ to 76° 53′71″E longitude covering total geographical area of about 1,818 km^2^. The mean annual rainfall of Bharmour is 1500 mm, and the mean annual temperature lies between 3°C to 30°C. Dharamshala lies in between 800 m to 1500 m amsl and extends 31°21′2″ to 32°59′25″N latitude and 75˚47′55″ to 77°45′72″E longitude. Dharamshala falls under wet temperate zone where the annual mean temperature is about 19.1 ± 0.5°C and annual rainfall is about 2900 ± 639 mm, as reported by 1951–2010 data.

In order to study wild edible plants used by the Gaddis, a questionnaire was prepared and used as a tool for the collection of information by personal interview. The questionnaire was divided into two parts: demographic and ethnobotanical data. The first part deals with the personal information of Gaddis like name, age, sex, occupation, etc., while the second part contained questions related to wild plant resource being utilized. Information on wild edible plants was collected from the Bharmour region of District Chamba from August 2017 to October 2017 and June 2018 to July 2018 when the Gaddis were on the way of their migration. Similarly, from Dharamshala, information was collected from March 2018 to April 2018 when the Gaddis were migrating from Khanyara. Key informants (Gaddis) were randomly selected as they were on their route of migration. Bharmour and Dharamshala were selected as study sites because the Gaddis reside in Bharmour in summer season and winters in Dharamshala. The 60 interviewed informants were made up of 48 male and 12 females, with age group between 20 and 65 years. The utilization of plant resources was identified through both the questionnaire and participatory techniques. Plant species are collected which are used by Gaddis as food. Collected plant species were dried, pressed, and mounted on the Herbarium Sheets for identification. These collected plant species were identified from the BSI (Botanical Survey of India), Dehradun. The collected plant material were given voucher number from SUBMS/BOT-501 to SUBMS/BOT-519 and SUBMS/BOT-2051 to SUBMS/BOT-2080 and submitted the herbarium sheets in the Herbarium of Shoolini University, Solan.

## 3. Results and Discussion

**Figure 2:**
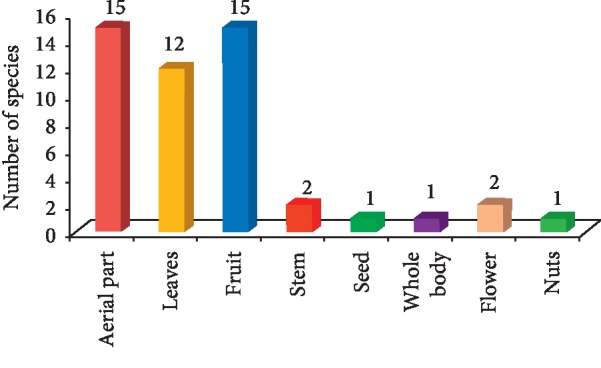
Number of species in which different plant parts were used by Gaddi tribe of Kangra and Chamba district of western Himalaya.

**Figure 3:**
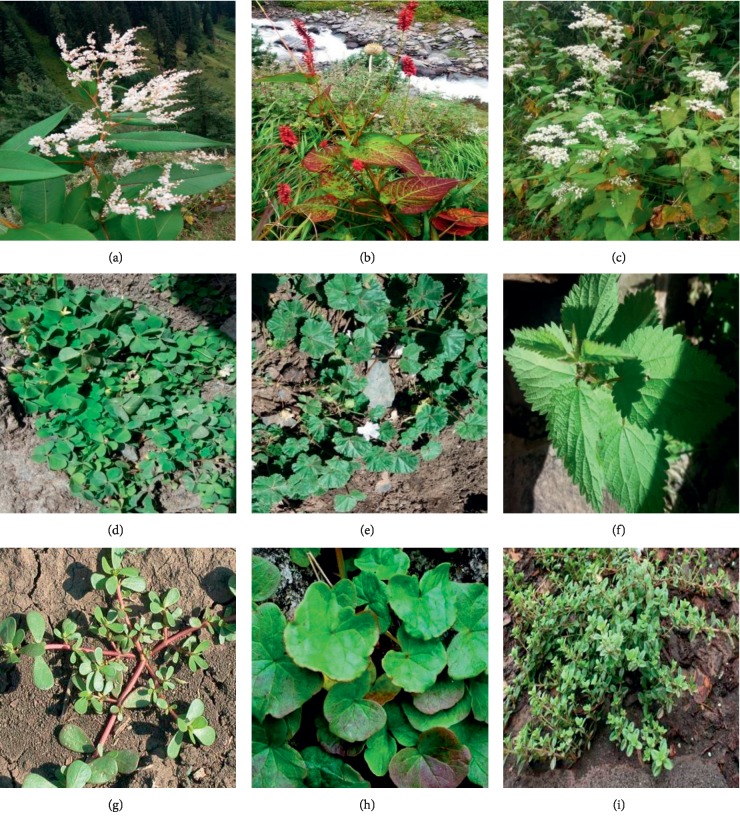
Wild edible plants: (a) *Polygonum polystachyum*, (b) *Polygonum hydropiper*, (c) *Fagopyrum esculentum*, (d) *Oxalis corniculata*, (e) *Malva verticellata*, (f) *Urtica hyperborea,* (g) *Portulacca oleracea,* (h) *Oxyria diygna*, and (i) *Thymus serphyllum.*

A total of 49 species were reported which were commonly consumed as vegetables, fruits, spices, chutney, etc. Among theses, 20 plants of 12 families were collected from the Bharmour region between August 2017 and October 2017 and are presented in Table 1. It is evident from Table 1 that nearly half of these plants were of Polygonaceae and Rosaceae families. It was also observed that out of 20 plants used by Gaddis from Bharmour region, fruits of 9 plants were consumed. Pickle and chutney form important food part in all the meals and is taken from *Oxalis corniculata* and *Oxyria digyna*. It is evident from Table 2 that 16 plants of 12 families were collected from the Bharmour region between June-July 2018. Among these *Viburnum contifolium, Rubus niveus*, and *Ficus palmata* were used as fruit, while all others as vegetable. Mostly aerial parts like leaves and flowers were used as vegetables. A total of 13 plants of 11 families were collected from Khanyara, Dharamshala, Kangra district (Table 3). Overall number of species used and various plant parts consumed by the Gaddis are 15 species (aerial parts, fruits), 12 species (leaves), 2 species (stem, flower), and 1 species (seed, whole body, and nuts) as shown in Figure 2. Herbs made up the highest proportion of the edible species (29) followed by trees (10), shrubs (8), climber (1), and fungus (1). Images of some collected wild edible plants is shown in Figure 3 and these are (a) *Polygonum polystachyum*, (b) *Polygonum hydropiper* (c) *Fagopyrum esculentum*, (d) *Oxalis corniculata*, (e) *Malva verticellata*, (f) *Urtica hyperborea*, (g) *Portulaca oleracea*, (h) *Oxyria digyna*, and (i) *Thymus serphyllum*.

There are over 20,000 species of wild edible plants in the world [16] and 1532 edible wild food species reported in India [17], of which over 675 species grow in the Indian Himalayan region [18]. A total of 58 wild edible plant species were used by Gujjar and Bakerwal tribes of District Rajouri (J&K) [19]. 50 wild edible plants belonged to 33 families from the Kishtwar high-altitude national park in Northwest Himalaya [20]. These plants were consumed as fruit, vegetables, and flavouring agent [21]. From the Alaknanda Valley of Garhwal Himalaya, India, 55 plant species belonging to 35 families were recorded. These plant species were consumed raw and prepared in to vegetables [22]. Similarly from Chamba district of Himachal Pradesh, 50 plant species of which 23 are herbs, 13 were trees, 2 were climbers, and 12 shrubs were used as ethno medicinal [4]. 50 species were used as wild edible plants which are in shepherds' route from high hills to low hills in the Kinnaur district of Himachal Pradesh [2]. *Morchella esculenta* (fungi), reported by Radha and co-workers (2018), were found to be absent in our studies except in Bharmour region of Chamba district indicating their extinction from the region.

The present studies are in line with earlier studies as stated above. The Gaddis are poor people and are dependent on edible plants growing in forest area or common lands. The nutritional requirements, especially vitamins, proteins, etc., are met through wild edible plants. Sometimes, the tribes collect these fruits in bulk and sell them in market which forms an important source of cash earnings. These plants generally belong to Rosaceae, Moraceae, and Fabaceae families. However, due to over exploitation, overgrazing, climate change, etc. these species are declining and are at risk [19].

## 4. Conclusion

The present study showed that different wild plants were used as food by Gaddi community of western Himalaya in order to sustain their life. Plant parts used commonly were leaves, fruits, and stem. These edible plants provide food and nutrition such as essential amino acids, vitamins, and minerals for this community to stay healthy. Unfortunately, the traditional knowledge on the use of wild edible plants is vanishing due to the modernization, and there is a need to document the traditional knowledge associated with a particular tribe.

## Figures and Tables

**Table 1:** Wild edible plants used by Gaddi tribes during migration in August–October months from Bharmour region of District Chamba.

Family	Botanical name	Common name	Local name	Time of availability	Habit	Plant part and mode of use
Brassicaceae	*Alliaria petiolata* (M. Bieb.)	Palak	Jangli palak	June–September	Herb	Aerial part, used as vegetable
Cucurbitaceae	*Coccinia grandis* (L.) *Voigt*	Bankrela	Bankakaru	August–October	Climber	Fruit, used as fruit
Elaegnaceae	*Elaeagnus umbellata* Thunb.	Silver berry	Bheen	July–September	Shrub	Fruit, used as fruit
Juglandaceae	*Juglans regia* Linn.	Akhrot	Khod	July–September	Tree	Nuts, used as dry fruit
Lamiaceae	*Thymus serphyllum* Linn.	Ban-ajwain	Ban-ajwain	April–July	Herb	Aerial parts, used as spices
Malvaceae	*Malva verticillata.*L.	Sonchal	Sonchal	July–October	Herb	Aerial parts, used as vegetable
Oxalidaceae	*Oxalis corniculata* L.	Amrul	Ambi	May–September	Herb	Aerial parts, used as chutney
Polygonaceae	*Fagopyrum esculentum* Moench	Buckwheat	Fafru	June–October	Herb	Aerial parts, used as vegetable
	*Oxyria digyna* (L.) Hill	Mountain sorrel	Amblu	June–September	Herb	Aerial Parts, used as chutney
	*Polygonum hydropiper* L.	Marshpepper knotweed.	Lahoul tarodu	June–October	Herb	Leaves, used as vegetable
	*Polygonum polystachyum* Wall. ex Meisn.	Knotweed	Badi tatod	June–October	Herb	Leaves, used as vegetable
Portulacaceae	*Portulaca oleracea* L.	Purslane	Kulfa	March–September	Herb	Aerial parts, used as vegetable
Rosaceae	*Pyrus baccata* L.	Mountain ash	Khajju	August–October	Tree	Fruit, used as fruit
	*Prunus cornuta* (Wall. ex Royle) Steud.	Jamun	Jamu	July–October	Tree	Fruit, used as fruit
	*Prunus persica* (L.) Batsch	Aadu	Aadu	July–September	Tree	Fruit, used as fruit
	*Pyrus pashia* Buch.-Ham. ex D. Don	Himalayan pear	Kainth	July–September	Tree	Fruit, used as fruit
	*Rosa brunonii* Lindl.	Hipberries	Kreru	July–November	Shrub	Fruit, used as fruit
Rutaceae	*Zanthoxylum alatum* Roxb.	Tumbru	Tirmir	June–October	Shrub	Fruit, as fruit
Urticaceae	*Urtica hyperborea* Jacq. ex Wedd.	Bichubuti	Ain	June–October	Herb	Leaves, used as vegetable
	*Urtica dioica* L.	Bichubuti	Ain	June–September	Herb	Leaves, used as vegetable

**Table 2:** Wild edible plants used by Gaddi tribes during migration in June-July months from Bharmour region of district Chamba.

Family	Botanical name	Common name	Local name	Time of availability	Habit	Mode of use
Adoxaceae	*Viburnum cotinifolium* D.Don	Tustus	Tarandhole	June-July	Shrub or small tree	Fruit, used as fruit
Amaryllidaceae	*Allium rubellum* M.Bieb.	Lehsun	Jangli pyaj	July–September	Herb	Aerial part, used as vegetable
Berberidaceae	*Berberis chitria* Buch.-Ham. ex Lindl.	Kashmal	Nigghi	March–July	Shrub	Leaves, used as spices
Brassicaceae	*Capsella bursa* –*pastoris* (L.) Medik.	Shepherd's purse	Dharsaag	April–July	Herb	Aerial part, used as vegetable
Caryophyllaceae	*Stellaria aquatica* (L.) Scop.	Giant cheekweed	Kohn	April–July	Herb	Aerial part, used as vegetable
	*Stellaria monosperma* Buch.-Ham. ex D. Don	Cheekweed	Katri	April–July	Herb	Aerial part, used as vegetable
	*Silene vulgaris* (Moench) Garcke	Maidenstairs	Ghandli	April–July	Herb	Aerial part, used as vegetable
Chenopodiaceae	*Chenopodium album* Linn.	Bathu	Kunah	April–August	Herb	Leaves, used as vegetable
Lamiaceae	*Mentha piperita* Linn.	Mint	Pudeena	June–September	Herb	Aerial part, used as chutney
Malvaceae	*Malwa sylvestris* Linn.	Sonchal	Sonchal	April–September	Herb	Leaves, used as vegetable
Moraceae	*Ficus palmata* Forssk.	Fegda	Fakuda	March–June	Tree	Fruit, used as fruit
	*Morus serrata Roxb.*	Krum	Krooon	May–June	Tree	Fruit, as fruit
Morchellaceae	*Morchella esculentum*	Gucchi	Gucchi	March–June	Fungus	Whole plant, used as vegetable
Polygonaceae	*Fagopyrum dibotrys* (D. Don) H. Hara	Buchwheat	Fafru	May–October	Herb	Leaves, used as vegetable
	*Rheum emodii* Wall.	Chukri	Gandhol	May–July	Herb	Stem, used as chutney
Rosaceae	*Rubus niveus* Thunb.	Himalayan strawberry	Aakhein	May–July	Shrub	Fruit, used as fruit

**Table 3:** Wild edible plants used by Gaddi tribes during migration in March-April months from Khanyara region (Dharamshala) district Kangra.

Family	Botanical name	Common name	Local name	Time of availability	Habit	Mode of use
Aspidiaceae	*Diplazium esculentum* (Retz.) Sw.	Lingad	Kasrod	April–July	Fern	Aerial part, used as vegetable and pickle
Asparagaceae	*Asparagus gracilis* Royle.	Sparrow grass	Grass	March-April	Herb	Aerial part, used as vegetable and pickle
Caryophyllaceae	*Stellaria peniculata* Edgew.	Cheekweed	Saag	March-April	Herb	Aerial part, used as vegetable
Crucifereae	*Nasturtium montanum* Wall.	Water cress	Chunali	March-April	Herb	Aerial part, used as vegetable
Ericaceae	*Rhododendron arboreum* Sm.	Burans	Chhihu	February–April	Shrub or small tree	Flower, used as chutney
Fabaceae	*Bauhinia variegata* Linn.	Krali	Krali	February-March	Tree	Flower, used as vegetable and pickle
Lamiaceae	*Mentha arvensis* Linn.	Mint	Jangli pudeena	March–August	Herb	Aerial parts, used as chutney
Moraceae	*Ficus auriculata* Lour.	Triamblu	Triamblu	April–June	Herb	Aerial parts, used as chutney
	*Ficus palmata* Forssk.	Fegda	Fakuda	April–June	Tree	Fruit, used as fruit
Polygonaceae	*Rumex nepalensis* Spreng.	Albal	Albal	April–June	Herb	Leaves, used as vegetable
Rosaceae	*Fragaria indica* (Ander) Deolf.	Strawberry	Laddu	April–June	Herb	Fruit, used as fruit
	*Rubus ellipticus* Sm.	Himalayan strawberry	Aakhein	March–June	Shrub	Fruit, used as fruit
Urticaceae	*Gerardiana diversifolia* Linn.	Bichubuti	Ain	March–May	Herb	Leaves, used as vegetable

## Data Availability

Previously reported (Uses of plant Biodiversity among the Tribal communities of Pangi valley of District Chamba in cold desert Himalaya, India) data were used to support this study and are available at (DOI. http://www.doi.org/10.1155/2014/753289. Source: PubMed). These prior studies are cited at relevant places within the text as references (Pawan Kumar Rana,1,2; Puneet Kumar,1; Vijay Kumar Singhal,1; and Jai Chand Rana,2).
